# Inhibitory effect of Yukmijihwang-tang, a traditional herbal formula against testosterone-induced benign prostatic hyperplasia in rats

**DOI:** 10.1186/1472-6882-12-48

**Published:** 2012-04-20

**Authors:** In Sik Shin, Mee Young Lee, Hye Kyung Ha, Chang Seob Seo, Hyeun-Kyoo Shin

**Affiliations:** 1 Basic Herbal Medicine Research Group, Korea Institute of Oriental Medicine, 483 Expo-ro, Yusung-gu, Daejeon 305-811, Republic of Korea; 2 College of Veterinary Medicine, Chonnam National University, Gwangju, 500-757, Republic of Korea

**Keywords:** Yukmijihwang-tang, Traditional herbal formula, Benign prostatic hyperplasia, Dihydrotestosterone

## Abstract

**Background:**

Yukmijihwang-tang, a traditional herbal formula, has been used for treating disorder, diabetic mellitus and neurosis in China (Liu-wei-di-huang-tang in Chinese), Japan (Lokumijio-to in Japanese) and Korea for many years. In this study, we investigated the effects of Yukmijihwang-tang water extract (YJT) on the development of benign prostatic hyperplasia (BPH) using a rat model of testosterone propionate (TP)-induced BPH.

**Methods:**

A total of 30 rats were divided into five groups. One group was used as a control and the other groups received subcutaneous injections of TP for 4 weeks to induce BPH. YJT (200 or 400 mg/kg) was administered daily for 4 weeks to two groups by oral gavage concurrently with the TP. The animals were euthanized, the prostate and body weights were recorded, and tissues were subjected to hormone assays and histomorphology. In addition, we investigated proliferating cell nuclear antigen (PCNA) expression in the prostate using immunoblotting.

**Results:**

Animals with BPH showed significantly increased absolute and relative prostate weights, increased dihydrotestosterone levels in the serum or prostate and increased PCNA expression in the prostate; however, YJT-treated animals showed significant reductions compared with the animals with TP-induced BPH. Histomorphology also showed that YJT inhibited TP-induced prostatic hyperplasia.

**Conclusions:**

These findings indicate that YJT effectively inhibited the development of BPH and might be a useful drug clinically.

## Background

Benign prostate hyperplasia (BPH) is a urological disorder caused by the noncancerous enlargement of the prostate as men age. As the prostate enlarges, it can constrict the urethra, inducing various symptoms including a weak urinary stream, incomplete bladder emptying, nocturia, dysuria and bladder outlet obstruction [1,2]. These symptoms associated with BPH are known as lower urinary tract symptoms (LUTS) [3]. Currently, the two main medications used for treatment of BPH are α_1_-adrenergic receptor antagonists and 5α-reductase inhibitors [4]. The α_1_-adrenergic receptor antagonists, including doxazosin, terazosin and tamsulosin, are the initial drugs for treating BPH, and they alleviate LUTS by relaxation of smooth muscle in the prostate and the neck of the bladder [5,6]. On the other hand, the 5α-reductase inhibitors inhibit the development of BPH via a reduction in dihydrotestosterone (DHT) production [7]. In the development of BPH, 5α-reductase catalyzes the conversion of testosterone to DHT, which induces an increase in the DHT level in the prostate. This accelerates hyperplasia of the stromal and epithelial cells of the prostate, resulting in prostatic enlargement [8]. As mentioned above, α_1_-adrenergic receptor antagonists and 5α-reductase inhibitors are effective in treating men with BPH. However, these drugs are limited because of their side effects, including decreased libido, ejaculatory or erectile dysfunction, and nasal congestion [9,10].

Yukmijihwang-tang (Liu-wei-di-huang-tang in Chinese; Lokumijio-to in Japanese), an oriental herbal formula, has been used for many years in Korea, China and Japan. It is composed of six herbs: *Rehmannia glutinosa Cornus officinalis Dioscorea batatas Paeonia suffruticosa Poria cocos Alisma orientale *. In particular, Yukmijihwang-tang is one of the most widely used herbal formulas in Korea and China. A Recent report showed that Yukmijihwang-tang was ranked first in consumption in the Korean herbal medicine market [11]. In China, the annual market turnover of Yukmijihwang-tang in herbal medicine is about US$633 million [12]. Traditionally, Yukmijihwang-tang is applied in treating renal disorders, diabetes mellitus, and neurosis [13]. There have been many studies on the pharmacological effects of Yukmijihwang-tang, such as protection against renal ischemia/reperfusion [14], memory enhancement [15], stimulation of spermatogenesis [16], inhibition of bone loss [17], anti-asthmatic effects [18] and antidiabetic effects [13]. Although many researchers have investigated the pharmacological effects of Yukmijihwang-tang, there has been no study on its possible protective effects against BPH.

Therefore, this study investigated the effects of an aqueous extract of Yukmijihwang-tang (YJT) on testosterone-induced BPH in rats by measuring prostate weight changes and the DHT levels in the serum and prostate, by western immunoblotting and histomorphology.

## Methods

### Preparation of Yukmijihwang-tang

The Yukmijihwang-tang formula was prepared in our laboratory from a mixture of chopped crude herbs purchased from Omniherb (Yeongcheon, Korea) and HMAX (Chungbuk, Korea). Before performing the study, identity of each crude herb was confirmed by Professor Je-Hyun Lee at the Oriental College of Dongguk University (Gyeongju, Korea). Yukmijihwang-tang was prepared as described in Table 1 and extracted in distilled water at 100°C for 2 h. The extract was then evaporated to dryness and freeze-dried (yield: 27.0%). An analysis of the chemical contents of YJT was conducted using high performance liquid chromatography (HPLC) system in our previous study [12]. The chemical standards used to identify and quantitate compounds in the YJT included the following: 5-hydroxymethyl-2-furaldehyde (5-HMF) as a component of *Rehmannia glutinosa *, loganin of *Cornus officinalis *, and paeoniflorin and paeonol of *Paeonia suffruticosa *. The concentration of chemicals in YJT were measured as the following: 5-HMF 3.70 ± 0.11 mg/g, loganin 1.77 ± 0.05 mg/g, paeoniflorin 1.08 ± 0.03 mg/g, and paeonol 1.98 ± 0.02 mg/g.

**Table 1:** Composition of YJT

**Scientific name**	**Amount (g)**	**Company of purchase**	**Source**
*Rehmannia glutinosa *	8.0	Omniherb	Kunwi, Korea
*Cornus officinalis *	4.0	Omniherb	Gurye, Korea
*Dioscorea batatas *	4.0	Omniherb	Kunwi, Korea
*Paeonia suffruticosa *	3.0	HMAX	China
*Poria cocos *	3.0	Omniherb	Yeongcheon, Korea
*Alisma orientale *	3.0	Omniherb	Imsil, Korea
Total	25.0		

### Animals

Male 12-week-old Wistar rats (n = 30) weighing 250 – 350 g (Central Lab. Animal. Inc., Seoul, Korea) were housed in a room maintained at 18–23°C and at a relative humidity of 40–60% with an alternating 12 /12 h light/dark cycle. They were offered a standard laboratory diet and water *ad libitum *. All experimental procedures were carried out in accordance with the NIH Guidelines for the Care and Use of Laboratory Animals and were approved by Korea Institute of Oriental Medicine Institutional Animal Care and Use Committee. The animals were cared for in accordance with the dictates of the National Animal Welfare Law of Korea.

### Experimental procedures

BPH was induced by subcutaneous injection of testosterone propionate (TP, 3 mg/kg, Tokyo Chemical Ins. Co., Tokyo, Japan) for 4 weeks. After 1 week of acclimatization, the rats were divided into five groups: (A) a normal control group that received phosphate-buffered saline (PBS, p.o.) with corn oil (s.c.); (B) a BPH group that received PBS (p.o.) with TP (s.c.); (C) a positive control group that received finasteride (10 mg/kg, p.o.) with TP (s.c.); and (D and E) YJT groups that received YJT at 200 or 400 mg/kg (p.o.), respectively, with TP (s.c.). Finasteride, a 5α-reductase inhibitor, was used as a positive anti-BPH drug and was purchased from Sigma-Aldrich (St Louis, MO, USA). Its effective dose for treating BPH was determined based on a previous study [19]. All materials were administered to animals once daily for 4 weeks, and body weight was measured weekly. The application volumes were 5 mL/kg for oral administration (PBS, finasteride and YJT) and 3 mL/kg for subcutaneous injection (corn oil and TP) and were calculated in advance based on the most recently recorded body weights of individual animals. After the last treatment, all animals were fasted overnight and euthanized using pentobarbital at 100 mg/kg body weight injected intraperitoneally (Han Lim Pharmaceutical. Co. Ltd., Yongin, Korea). Blood samples were drawn from the caudal vena cava, and the serum was separated by centrifugation. Serum was stored at at −80°C for hormone assays. The prostates were removed immediately and weighed. Relative prostate weight was calculated as the ratio of prostate weight to body weight. The percentage inhibition of the increase in prostate weight induced by YJT was determined according to previous study [20]. The ventral lobe of the prostate was divided in half. One half was fixed using 10% neutral-buffered formalin and embedded in paraffin for histomorphology and the other was stored at −80°C for other analyses.

### Preparation of prostate homogenates

Prostatic tissue was homogenized (1/10 w/v) in tissue lysis/extraction reagent containing protease inhibitors (Sigma-Aldrich) using an IKA T10 Basic (IKA Works, Staufen, Germany). Homogenates were centrifuged at 12,000 *g * for 25 min at 4°C. Protein concentrations in the supernatant fractions were determined using Bradford reagent (Bio-Rad Laboratories, Inc., Hercules, CA, USA).

### Measurement of DHT levels in the serum and prostate

Levels of DHT in serum and the prostate were determined using an enzyme-linked immunosorbent assay (ELISA) kit according to the manufacturer’s instructions (ALPCO Diagnostics, Salem, NH, USA). The absorbance was measured at 450 nm using a microplate ELISA reader (Bio-Rad Laboratories, Inc.). Values are expressed per mg protein for the prostate and per mL for serum.

### Western blotting

Equal aliquots (30 μg) of total lung protein were heated at 100°C for 5 min then loaded onto 12% SDS−PAGE gels, followed by transfer to nitrocellulose membranes at 100 V for 2 h. The membranes were blocked for 1 h with Tris-buffered saline containing 0.05% Tween-20 (TBST) plus 5% skim milk and were incubated with anti-proliferating cell nuclear antigen (anti-PCNA, 1:1000 dilution; Santa Cruz Biotechnology, Santa Cruz, CA, USA) and anti-β-actin (1:1000 dilution; Cell Signaling Technology, Danvers, MA, USA) overnight at 4°C. The membranes were washed three times with TBST and then incubated with a 1:10,000 dilution of horseradish peroxidase-conjugated secondary antibody (Jackson ImmunoResearch, West Grove, PA, USA) for 1 h at room temperature. The membranes were again washed three times with TBST and were then developed using an enhanced chemiluminescence kit (Amersham Biosciences, Little Chalfont, UK). For quantitative anlaysis, band densities were determined using Chemi-Doc (Bio-Rad Laboratories, Inc.).

### Histomorphology

Fixed prostate tissue embedded in paraffin wax were cut into 4 μm thick sections and stained with hematoxylin (Sigma-Aldrich MHS-16) and eosin (Sigma-Aldrich HT110-1-32). The sections were mounted and coverslipped using mounting medium (Invitrogen, Carlsbad, CA, USA) and then examined under a microscope (Nikon, Tokyo, Japan). Measurement of prostate epithelial thickness was performed using an image analyzer (Molecular Devices Inc., CA, USA).

### Statistical analysis

Data are expressed as the means ± standard deviation (S.D.). Statistical significance was determined using analysis of variance. When tests showed a significant difference among groups, data were analyzed further using a multiple comparison procedure and Dunnett’s test. The significance levels were set at P < 0.05 and < 0.01.

## Results

### Effect of YJT on prostate weights

Rat in the BPH group showed absolute and relative prostate weights that were significantly greater than those of rats in the normal control group, whereas prostate weights in the finasteride-treated group were decreased markedly compared with the BPH group (Table 2). YJT-treated groups also exhibited significant decreases in absolute and relative prostate weights compared with the BPH group. In addition, YJT inhibited the TP-induced increase in prostate weight by 54.48% in the 200 mg/kg YJT group and by 50.79% in the 400 mg/kg YJT group. These results were similar to those for the finasteride-treated group. There were no significant differences in body weight changes among groups.

**Table 2:** Effects of YJT on body weights and prostate weights

**Groups**	**Prostate weights**		**% Inhibition**	**Body weights (g)**	
	**Absolute (g)**	**Relative (g)**		**Initial**	**Final**
NC	1.44 ± 0.10	0.35 ± 0.02		273.5 ± 8.50	416.7 ± 12.75
BPH	3.05 ± 0.55^##^	0.78 ± 0.15^##^		273.9 ± 8.94	391.5 ± 23.47
Finasteride	1.95 ± 0.28^**^	0.51 ± 0.04^**^	62.01%	276.0 ± 13.01	380.3 ± 46.58
YJT-200	2.14 ± 0.25^**^	0.54 ± 0.06^**^	54.48%	277.2 ± 13.08	394.2 ± 27.11
YJT-400	2.25 ± 0.27^**^	0.56 ± 0.10^**^	50.79%	278.9 ± 9.34	404.8 ± 26.01

NC: corn oil injection (s.c) + PBS (p.o.), BPH: testosterone (s.c) + PBS (p.o.), Finasteride: testosterone (s.c) + finasteride (10 mg/kg, p.o.), YJT-200 and −400: testosterone (s.c) + YJT (200 and 400 mg/kg, respectively, p.o.).

^##^*P * < 0.01 when compared with the NC group.

^**^P  < 0.01 when compared with the BPH group.

### Effects of YJT on DHT levels in serum

**Figure 1:**
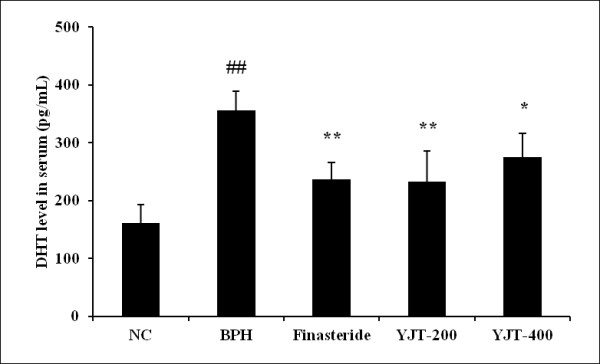
**Effects of YJT on DHT levels in the serum.** NC: corn oil (s.c.) + PBS (p.o), BPH: testosterone (s.c.) + PBS (p.o.), Fin: testosterone (s.c.) + finasteride (10 mg/kg, p.o.), YJT-200, 400: testosterone (s.c.) + YJT (200 or 400 mg/kg, respectively, p.o). ^##^Significant difference at *p * < 0.01 compared with the NC group. ^*^,^**^Significant difference at *P * < 0.05 and *P * < 0.01compared with the BPH group, respectively.

The BPH group showed a significant increase in serum DHT level (356.5 ± 33.30 pg/mL, *P * < 0.01) compared with the normal control group (161.38 ± 32.09 pg/mL; Figure 1). In contrast, the finasteride-treated group showed a significantly reduced serum DHT level (236.6 ± 29.88 pg/mL, *P * < 0.01) compared with the BPH group. Similarly to finasteride-treated group, the YJT-treated groups showed significant reduction in DHT levels (233.4 ± 52.53 pg/mL in the 200 mg/kg group, *P * < 0.01; 275.3 ± 40.79 pg/mL in the 400 mg/kg group, *P * < 0.05) compared with the BPH group.

### Effects of YJT on DHT levels in the prostate

**Figure 2:**
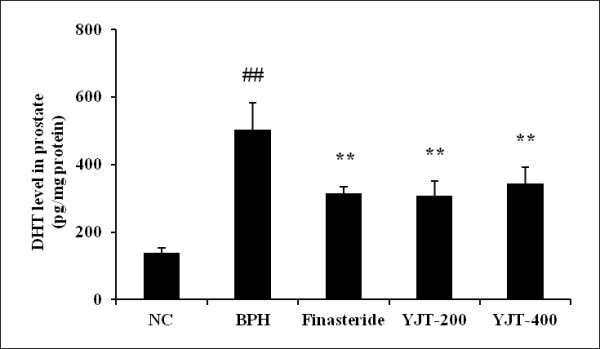
**Effects of YJT on the DHT levels in the prostate.** NC: corn oil (s.c.) + PBS (p.o), BPH: testosterone (s.c.) + PBS (p.o.), Fin: testosterone (s.c.) + finasteride (10 mg/kg, p.o.), YJT-200, 400: testosterone (s.c.) + YJT (200 or 400 mg/kg, respectively, p.o). ^##^Significant difference at *P * < 0.01 compared with the NC group. ^**^Significant difference at *P * < 0.01 compared with the BPH group.

The DHT level in the prostates of the BPH group (547.6 ± 140.84 pg/mg protein, *P * < 0.01) was markedly higher than in the negative normal controls (Figure 2). However, prostatic DHT level in the finasteride-treated group (315.5 ± 17.98 pg/mg protein, *P * < 0.01) was significantly lower than in the BPH group. Prostatic DHT levels in the YJT-treated rats were 308.0 ± 42.93 pg/mg protein in the 200 mg/kg group and 343.6 ± 49.57 pg/mL in the 400 mg/kg group, which were significantly less than the level in the BPH group.

### Effects of YJT on PCNA expression in the prostate

**Figure 3:**
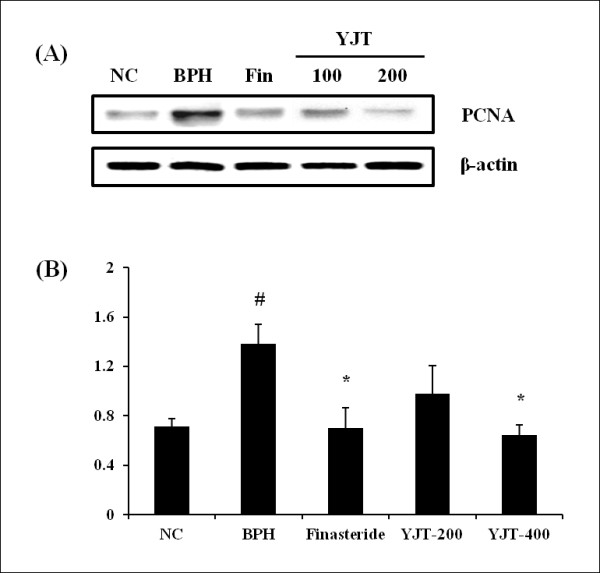
**Effects of YJT on the expression of PCNA protein. (A)** Image on the gel, **(B)** Relative units of PCNA expression (ratio of PCNA to β-actin). NC: corn oil (s.c.) + PBS (p.o), BPH: testosterone (s.c.) + PBS (p.o.), Fin: testosterone (s.c.) + finasteride (10 mg/kg, p.o.), YJT-200, 400: testosterone (s.c.) + YJT (200 or 400 mg/kg, respectively, p.o). ^#^Significant difference at *p * < 0.05 compared with the NC group. ^*^Significant difference at *P * < 0.05 compared with the BPH group.

The expression of PCNA protein increased in the BPH group compared with the normal control group and decreased in the finasteride-treated group compared with the BPH group. Expression of PCNA protein was also reduced in the YJT-treated groups compared with the BPH group (Figure 3A , B).

### Effects of YJT on prostatic epithelial hyperplasia

**Figure 4:**
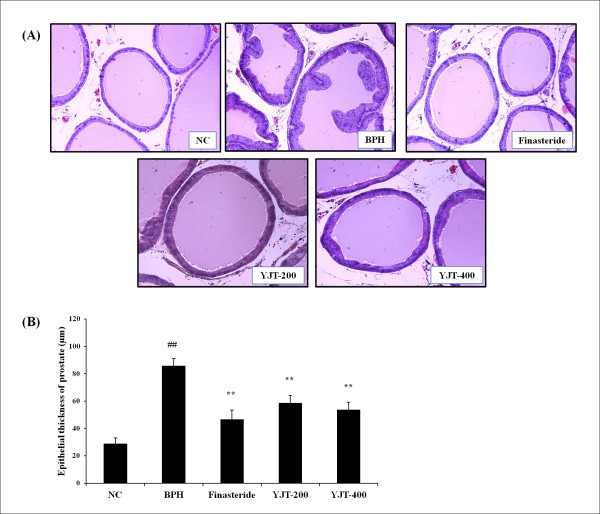
**Effects of YJT on prostatic hyperplasia. (A)** Histomorphological changes of prostate, **(B)** Prostatic epithelial thickness. NC: corn oil (s.c.) + PBS (p.o), BPH: testosterone (s.c.) + PBS (p.o.), Fin: testosterone (s.c.) + finasteride (10 mg/kg, p.o.), YJT-200, 400: testosterone (s.c.) + YJT (200 or 400 mg/kg, respectively, p.o). ^##^Significant difference at *P * < 0.01 compared with the NC group. ^**^Significant difference at *P * < 0.01 compared with the BPH group.

The BPH group showed prostatic epithelial hyperplasia (Figure 4A). Finasteride-treated animals showed mild epithelial hyperplasia compared with the BPH animals. YJT-treated animals also showed a reduction in epithelial hyperplasia compared with BPH animals, which was similar to the reduction in finasteride-treated animals. The BPH group showed significantly increased prostatic epithelial thickness compared with the negative control group; however, the YJT-treated groups and the finasteride-treated group showed markedly reduced hyperplasia compared with the BPH group (Figure 4B).

## Discussion

We evaluated the inhibitory effects of YJT on the development of BPH using TP-induced BPH in rats. Animals with BPH induced by TP treatment showed significant increases in absolute and relative prostate weights, increased DHT levels in the serum and prostate and elevated prostatic epithelial hyperplasia with elevated PCNA expression. In contrast, the YJT-treated animals showed significant reductions in absolute and relative prostate weights and in DHT levels in the serum and prostate, and mild prostatic epithelial hyperplasia with decreased PCNA expression.

Rats with BPH showed significantly increased absolute and relative prostate weight compared with the negative control animals; however, YJT-treated animals showed significant reductions in these measures compared with the BPH animals. According to previous studies, increased prostate weight is an important marker indicating the development of BPH [20,21]. BPH involves epithelial and stromal hyperplasia of the prostate, resulting in an increase in the prostate weight. When sufficiently large, the prostate constricts the urethral canal to cause partial or sometimes complete obstruction [2]. For these reason, many studies have tested the inhibitory effects of various substances on the development of BPH by measuring prostate weights [22,23]. In the results from histomorphology of the prostate, the rats with BPH showed epithelial hyperplasia with an increase in epithelial thickness compared with the negative control animals. In contrast, the YJT-treated rats showed mild prostatic epithelial hyperplasia with a reduction in epithelial thickness compared with BPH animals. These results are in agreement with the prostate weight results. In addition, we evaluated PCNA expression in the prostate to test the inhibitory effects of YJT on epithelial cell proliferation in rats with TP-induced BPH. Measuring PCNA is commonly used to evaluate cellular proliferation in benign and malignant proliferating tissues, as the PCNA level is correlated directly with the degree of proliferation [24,25]. Previous studies on BPH demonstrated that PCNA is a meaningful indicator of prostatic proliferation, and that its expression in the prostate is significantly increased in animals with experimentally induced BPH [26,27]. In the present study, YJT significantly decreased PCNA expression in the prostate compared with the BPH group, in parallel with a reduction in the prostatic hyperplasia. Thus, YJT treatment effectively inhibited the prostatic hyperplasia induced by TP.

DHT, a steroid hormone produced from testosterone by the enzyme 5α-reductase, is the primary active metabolite of testosterone [28]. The role of DHT in BPH is well known, as it is the androgen responsible for prostate growth [8,29]. Because DHT has a 10 times higher affinity for the androgen receptor than testosterone, DHT easily binds to androgen receptors, which stimulates the transcription of growth factors that are mitogenic for the epithelial and stromal cells of the prostate [30]. Therefore, DHT is ultimately responsible for prostatic epithelial and stromal cell hyperplasia [31]. As mentioned above, because DHT is formed from testosterone by 5α-reductase, many studies have focused on reducing the DHT level by inhibiting this enzyme. Finasteride, a 5α-reductase inhibitor, is used as an elective drug for treating human BPH [8]. In previous studies, finasteride reduced DHT levels in the serum and prostate, inhibiting prostate enlargement and attenuating the LUTS caused by BPH [32,33]. However, there is limited clinical use of 5α-reductase inhibitors including dutasteride and finasteride, because of their adverse effects [9,10]. In the present study, YJT-treated animals showed significant reductions in DHT levels of the serum and prostate compared with the BPH animals, as did the finasteride-treated rats. These results were consistent with the changes in prostate weights, PCNA expression levels and histomorphology. These findings indicate that YJT suppressed the development of BPH in this animal model, in a manner closely associated with reductions in DHT levels. In addition, YJT has been proved to be safe through toxicity studies [11,12]. In particular, there was no observed adverse effect from YJT at up to 2000 mg/kg in acute and subchronic toxicity studies [12].

## Conclusion

In conclusion, YJT significantly reduced prostate weights, prostatic hyperplasia, PCNA expression, and DHT levels in the serum and prostates of experimental rats. These results indicate that YJT effectively inhibits the development of BPH. Combined with the proven safety of YJT, these findings strongly support the feasibility of using YJT therapeutically in treating BPH.

## Competing interest

The authors declare that they have no competing interests.

## Authors’ contributions

ISS, MYL and HKS participated in the design of the study data analyses and manuscript preparation. ISS and HH conducted the assays and analyses. CSS provided YJT samples. All authors read and approved the final manuscript.

## Pre-publication history

The pre-publication history for this paper can be accessed here:

http://www.biomedcentral.com/1472-6882/12/48/prepub
